# The Preoperative Level of Pain Predicts Chronic Pain in Patients Operated on for Degenerative Disc Disease—A Prospective Study

**DOI:** 10.3390/jcm14103467

**Published:** 2025-05-15

**Authors:** Agnieszka Pawełczyk, Rusłan Jekimov, Weronika Lusa, Redwan Jabbar, Katarzyna Kruzerowska, Tomasz Pawełczyk, Maciej Radek

**Affiliations:** 1 Department of Neurosurgery, Spine and Peripheral Nerves Surgery, Medical University of Lodz, 90-549 Lodz, Poland; agnieszka.pawelczyk@umed.lodz.pl (A.P.); jekrus@interia.pl (R.J.); rredwanbakal@gmail.com (R.J.); k.ceranowicz@onet.pl (K.K.); maciej.radek@umed.lodz.pl (M.R.); 2 Department of Affective and Psychotic Disorders, Medical University of Lodz, 92-216 Lodz, Poland; tomasz.pawelczyk@umed.lodz.pl

**Keywords:** degenerative disc disease, spine surgery, neurosurgery, postoperative pain, chronic pain, pain predictors, Visual Analogue Scale

## Abstract

**Background:** Postoperative pain is an unpleasant experience for the patient and impairs postoperative functional outcomes. The current literature on the influence of preoperative predictors on postoperative pain outcomes remains limited. This study aimed to identify sociodemographic, clinical, psychological, and temperamental predictors of postoperative pain in patients undergoing surgery for degenerative disc disease (DDD). **Methods**: Eighty-one adults with DDD, qualified for neurosurgical intervention, were enrolled. All patients underwent neurological and psychiatric evaluations, as well as preoperative pain assessments using the Visual Analogue Scale (VAS) and the West Haven-Yale Multidimensional Pain Inventory (WHYMPI). Psychological assessments included the Perceived Stress Scale, Hospital Anxiety and Depression Scale, Somatic Symptom Scale, temperament, and personality inventories (e.g., FCB-TI, NEO-FFI), and cognitive tests (Trail Making Test, Digit Span Test). Postoperative pain was re-evaluated with the VAS 12 weeks after surgery. Data were analyzed using univariate and multivariate statistical methods. **Results**: Univariate analyses revealed significant differences between the defined groups regarding lack of improvement of pain 12 weeks after surgery compared to preoperative VAS, systolic blood pressure, and four scales from the WHYMPI. However, stepwise logistic regression identified only preoperative VAS score as an independent predictor of postoperative pain improvement. Receiver Operating Characteristic analysis and Youden’s index indicated a preoperative VAS cut-off score of 6 as the most predictive. **Conclusions**: A VAS score of 6 or more before surgery independently predicts the absence of chronic pain 12 weeks postoperatively for patients without neurological deficits. Moreover, given the complexity of this topic, further prospective, randomized controlled research is essential.

## 1. Introduction

Pain is a complex and multifactorial phenomenon requiring a multidisciplinary approach that starts from the preoperative setting and extends well into patient recovery [1]. Pain sensitivity and perception are influenced and stimulated by various factors, including physical, psychological, and behavioral patterns, and any changes to any of these components can lead to differences in a range of pain sensations and responses to pain-management interventions. Unidentified pain following spinal surgery without underlying causes remains a major clinical challenge [2].

Despite the latest advances in neurosurgery and spine surgery involving developments in prosthesis, cutting-edge technology and surgical techniques, postoperative pain remains a significant factor affecting patient recovery and overall satisfaction. Postoperative pain following a spinal procedure for degenerative disc disease (DDD) not only causes psychosocial distress and an unpleasant experience for the patients but also impairs postoperative functional outcomes. The vast majority of surgical patients who undergo such surgery experience acute postoperative pain, with approximately 75% reporting various degrees of pain severity ranging from moderate to severe [3]. This represents a major challenge as insufficient postoperative pain control can lead to adverse physiologic effects among patients, including increased risk of opioid misuse, poor functional recovery, post-surgical complications, and the development of chronic pain [4]. Other studies have demonstrated that persistent postoperative pain affects 2–10% of adult patients, emphasizing the need for a definitive level of pain surveillance protocols or guidelines to adjust therapy and enhance therapeutic efficiency accordingly [2].

Recognizing pain sensitivity will always be crucial, both for the patients undergoing surgery and the treating surgeon. The current literature is scarce regarding the significance of preoperative pain predictors and consists of only a few systematic reviews [5,6,7], meta-analyses [7], and retrospective studies [8,9,10,11,12] that highlight the importance of predictive factors on postoperative pain.

Therefore, the present prospective study was designed to evaluate the predictive value of sociodemographic, psychological and clinical traits and their influence on postoperative pain in a cohort of patients operated on for degenerative disc disease (DDD). Moreover, we hypothesize that the results of the present study will enable a more accurate identification of the factors influencing the qualification of patients with pain symptoms associated with DDD for surgical intervention. In addition, the described study may contribute to establishing a more engaging and enhanced basis for further collaboration among neurosurgeons, psychologists, physiotherapists, and nurses to provide a better quality of care in managing postoperative pain among surgical patients. Collaborative efforts and a multidisciplinary approach can ensure comprehensive care that can address and navigate the complexities of postoperative pain management and ultimately contribute to better patient care and functional outcomes.

## 2. Materials and Methods

### 2.1. Participants

Eighty-one participants were enrolled in the study. The members of the study group were over 18 years old, mentally stable (without acute mental condition), experiencing pain due to degenerative disc disease (DDD) and accepted being treated by surgical intervention. The exclusion criteria were as follows: a history of neurological or chronic somatic disorders associated with pain experience (i.e., oncological, or rheumatoid diseases), head injury, alcohol or substance abuse or dependence, and psychiatric disease (schizophrenia, major depressive episode, bipolar disorder). All participants were Polish and Caucasian. Demographic and clinical information for all participants can be found in Table 1, Tables S1 and S2 (Supplementary Materials).

### 2.2. Procedure

All participants were tested during a single session with a neurosurgeon, psychiatrist, and neuropsychologist the day before surgery. All received a standard neurological and psychiatric examination.

The level of pain was evaluated with the Visual Analogue Scale (VAS) and with the West Haven-Yale Multidimensional Pain Inventory (WHYMPI) [13]. The WHYMPI is a 52-item, 12-scale inventory that is divided into three parts. Part I includes five scales designed to measure dimensions of chronic pain, including 1. perceived interference of pain in vocational, social/recreational, and family/marital functioning, 2. support or concern from a spouse or significant other, 3. pain severity, 4. perceived life control, and 5. affective distress. Part II assesses patients’ perceptions of the degree to which spouses or significant others display Solicitous, Distracting or Negative responses to their pain behaviors and complaints. Part III assesses patients’ reports of the frequency with which they engage in four categories of common everyday activities: Household Chores, Outdoor Work, Activities Away from Home, and Social Activities. WHYMPI was translated into Polish with the permission of the author, Professor Kerns.

The stress level was assessed with a Perceived Stress Scale (PSS-10) [14]—a 10-item questionnaire measuring an individual’s perceived stress levels [15].

The level of depression and anxiety was evaluated with the Hospital Anxiety and Depression Scale (HADS) [16,17] based on a 14-item self-report rating scale consisting of two subscales: HADS-A (anxiety) and HADS-D (depression).

The somatic symptom burden was assessed using a valid, 8-item self-report tool—Somatic Symptom Scale (SSS-8) [18,19].

Temperament was assessed with the Formal Characteristics of Behavior–Temperament Inventory (FCB-TI), an inventory based on the Regulative Theory of Temperament (RTT) by Strelau [20]. The FCB-TI is used for measuring temperament traits, which are defined as basic, primary, biologically determined personality measurements. It contains six subscales: Briskness, Perseverance, Sensory Sensitivity, Emotional Reactivity, Endurance, and Activity [21].

The personality of the participants was assessed with NEO- Five-Factor Inventory (NEO-FFI) [22,23]. It consists of 60 items, categorized into five dimensions: Neuroticism, Extraversion, Openness to Experience, Agreeableness, and Conscientiousness [24].

The Trail Making Test (TMT) is used for cognitive function evaluation. TMT comprises two parts: the TMT-A with visual scanning, processing speed and psychomotor speed assessment, and the TMT-B, which constitutes an important tool for cognitive flexibility, set-shifting and working memory measurement [25,26].

For the assessment of short-term and working memory, the Digit Span Test [27] was applied in two formats: Digit Span Forwards (DSF) and Digit Span Backwards (DSB) [28].

In the final part of the study, the patients indicated their pain experiences 12 weeks following the surgical intervention.

All participants gave their informed consent before inclusion in the study. The study received approval from the Ethical Committee of the Medical University of Lodz (No RNN/259/19/KE) and was performed according to the ethical standards laid down in the Declaration of Helsinki.

### 2.3. Statistical Analysis

The results collected as described above were subjected to statistical analysis. Both descriptive statistics and statistical inference methods were used. Both univariate and multivariate analyses were carried out. The arithmetic mean and median were calculated to measure the central tendency for the quantitative traits. The total range of variation of a given trait was presented using minimum and maximum values; the interquartile range, i.e., the difference between the values of the third and first quartiles, was calculated. The standard deviation was used as a measure of dispersion. Compliance with the assumed normal distribution was assessed using the Shapiro–Wilk test.

The significance of the differences in the studied variables between analyzed groups was analyzed using appropriate tests. For traits expressed on a nominal scale, the Chi2 test of independence was used, considering the appropriate number of degrees of freedom. The Chi2 independence test with Yates correction was used for low counts in the contingency tables. The significance level of the test, which determines the maximum probability of making an error of the first type (i.e., rejecting the null hypothesis due to random convergence giving the impression of a real relationship), was taken as α = 0.05. Two-tailed tests were used in the analyses. For quantitative variables, the Student’s *t*-test or the Mann–Whitney U-test was used, depending on the status of their assumptions.

Variables significant at α = 0.05 were then included in multivariate analyses to assess the presence of relationships between variables while controlling for the effect of intercorrelation. Multivariate analysis was carried out to determine the internal structure of the collected data and the actual role of the variables under study and to control for possible confounding variables.

Logistic regression was used to detect potential correlations and distinguish them from objective, independent risk factors for a lack of improvement in pain 12 weeks after the surgical procedure. Variables that demonstrated statistical significance in the univariate analyses were included as explanatory variables. Confidence intervals of 95% were adopted. Conditional stepwise forward logistic regression was used in the analyses. Cutoff points between risk categories for lack of pain improvement 12 weeks after the surgical procedure were then estimated using Receiver Operating Characteristics (ROC) analysis. based on the results, the J Youden statistic was calculated to identify the cutoff point while maximizing the sensitivity and specificity of the test [29].

The collected data were coded in Microsoft Excel. Statistical analyses were performed using JASP statistical software (JASP Team (2024). JASP (Version 0.18.3) [Computer software]. Available at: https://jasp-stats.org/ (accessed on 1 February 2025)) and Jamovi (Jamovi Project (2024). Jamovi (Version 2.3.28.0) [Computer software]. Retrieved from https://www.jamovi.org (accessed on 1 February 2025)).

## 3. Results

### 3.1. Characteristics of the Study Population

The characteristics of the study population are provided in Table 1 (qualitative variables), Tables S1 and S2 (quantitative variables). Tables S1 and S2 (both in Supplementary Materials) include the assessment of the degree of normality of the qualitative variables.

### 3.2. Univariate Analyses

Univariate analyses indicated that the defined groups demonstrated significant differences in the following continuous variables regarding lack of improvement of pain 12 weeks after the surgical procedure: preoperative VAS, systolic blood pressure (SBP), WHYMPI—Support, WHYMPI—Pain Severity, WHYMPI—Solicitous Responses and WHYMPI—Distracting Responses.

The effect size (Cohen’s d) for the significant differences ranged from low (0.276 for WHYMPI–Support) to moderate (0.651 for preoperative VAS). The results are shown in Table 2 and Table 3.

For the Student’s *t*-test, the effect size (ES) is determined by Cohen’s d. For the Mann–Whitney U Test, the effect size (ES) is determined by the rank-order correlation. Effect size was given when significant differences were present. Significant differences were underlined.

### 3.3. Multivariate Analyses

#### 3.3.1. Logistic Regression

All the variables that significantly differentiated the study groups in the univariate analysis were included in stepwise forward logistic regression.

The analyses revealed two models that identified independent risk factors for lack of pain improvement 12 weeks after the surgical procedure. The first model is composed of preoperative VAS, and the second model is composed of preoperative VAS and WHYMPI—Pain Severity, which are both measures of the severity of pain. The inclusion of WHYMPI—Pain Severity to the model already composed of preoperative VAS was not significant and did not increase the model fit (ΔChi^2^ = 3.703; *p* = 0.054); in addition, the WHYMPI—Pain Severity parameter in the second model was not significant (Wald test *p* = 0.081).

Because of the clinical similarities between the two variables included in the analysis (preoperative VAS and WHYMPI—Pain Severity), i.e., both measuring the intensity of pain but using different scales and the lack of significance of the WHYMPI—Pain Severity parameter in the second model, it was decided to accept the model composed of only preoperative VAS; this can be interpreted as an independent predictor of pain improvement after 12 weeks since the surgical procedure. The results of logistic regression analyses are shown in Table 4 and Table 5.

#### 3.3.2. Cut-Off Scores for a Preoperative VAS

The most efficient cut-off scores for a preoperative VAS and differentiating between the groups of patients that demonstrated the improvement of pain 12 weeks after the surgical procedure were calculated based on Receiver Operating Characteristics (ROC). Following this, J Youden’s index was calculated for different preoperative VAS cut-off scores. J Youden’s index allows the cut-off point to be identified while maximizing the sensitivity and specificity of a test. This analysis identified the highest area under the ROC curve (AUC) for a cut-off of six points for a preoperative VAS. The results of the ROC curve and Youden index calculations are shown in Table 6.

## 4. Discussion

The present study analyzes the predictive value of selected clinical, sociodemographic, personality, and temperamental variables regarding the persistence of pain 12 weeks after surgery in a cohort of 81 patients without any neurological deficits who had been diagnosed with degenerative disc disease (DDD) and operated on for pain. The results indicate that patients with persistent postoperative pain differed significantly from those without postoperative pain concerning certain variables assessed on admission. These differences included pain intensity measured on the VAS and the WHYMPI, systolic blood pressure (SBP), and a few parameters from the WHYMPI: perceived support from others, frequency of distracting responses, and frequency of solicitous responses. However, stepwise forward logistic regression analysis identified only preoperative pain intensity, measured by the VAS, as a statistically significant independent predictor of postoperative pain improvement.

Furthermore, our findings suggest that patients who experienced pain relief 12 weeks after spine surgery were characterized by preoperative pain intensity scores of 6 or higher on the VAS. The present study is one of the few to identify predictors of surgical treatment efficacy in patients undergoing procedures for pain associated with DDD and identify a VAS cutoff score that has predictive value.

Our findings also show that patients without chronic postoperative pain scored higher in the WHYMPI subscales: Support, Solicitous and Distracting Responses when compared to individuals with chronic pain. This suggests that people suffering from chronic pain received more support, protective and caring statements, and behavior from relatives. It may suggest that a supportive and solicitous relationship acts as a protective factor against persistent pain [30,31] affecting experienced stress and decreasing possible somatic responses. However, this conclusion needs further research.

Our data also suggest that the level of preoperative pain predicts postoperative pain improvement. These findings partially support those of prospective studies conducted by Kim et al. [32] and Angadi et al. [33], who observed that preoperative pain was a predictor of postoperative pain. Kim et al. found preoperative severity of back pain to be a predictive factor for acute back pain in a cohort of 117 patients who underwent surgery for lumbar spinal stenosis; Angadi et al. emphasized that not only preoperative pain level in VAS has predictive value, but heat pain threshold and HADS scores can also predict acute postoperative pain and analgesics requirements in 60 patients following lumbar fusion surgeries. However, our present observations better characterize chronic pain, as our data is based on a longer follow-up period (12 weeks post-procedure) than the previous studies (up to three days), which pertain more to acute pain.

Furthermore, a few retrospective studies in the literature align with our outcomes and highlight the predictive value of preoperative pain score in the surgical efficacy evaluation [9,12]. A study of 61 patients by Krutko et al. confirmed that preoperative neuropathic pain severity (assessed by DN4 questionnaire) is an independent predictor of surgical treatment efficacy in decompressive procedures for degenerative spinal stenosis, as evaluated by one-factor regression analysis [12]. However, Krutko et al. examined neuropathic pain using the DN4 questionnaire, whereas our present study employed the VAS to assess preoperative pain resulting from DDD.

Interestingly, Cook et al. reported that older age and higher baseline back pain with a VAS score greater than 6/10 predicted postoperative pain relief one year after the procedure in a cohort of 1108 patients [9]. These findings are consistent with our present data, which also indicate a VAS score >= 6 as a cut-off point. Nevertheless, a retrospective study of 310 individuals by Suzuki et al. based on multivariate stepwise logistic regression analysis found persistent low back pain following lumbar fusion surgeries to be associated with inter alia high preoperative pain level (VAS) and medical history of previous lumbar surgeries involving the decompression of neural structures [8]. Although this contrasts with our findings, Suzuki’s study is retrospective, and ours is prospective, and the evaluated groups differ in sample size and the length of the follow-up.

Our findings did not indicate that the tested demographic and clinical traits had any predictive value, which may suggest that chronic postoperative pain is not linked to them. Furthermore, these results are not fully consistent with the literature, as several investigators indicate that patient age, history of spine surgeries and smoking may predict postoperative pain [5,6,7,8,9,10,11]. However, our present findings were obtained by a prospective study based on conditional stepwise forward logistic regression, whereas the previous work included systematic reviews [5,6,7], meta-analysis [7], or retrospective studies [8,9,10,11,12]. A prospective study has many advantages over a retrospective study because it allows researchers to control data collection systematically, thereby reducing bias and enhancing the accuracy and reliability of results.

Our study also identified no predictive factors among the tested psychological measures. These results might suggest that personality and temperament traits, as well as psychopathological symptoms like depression and anxiety, do not predict postoperative pain. Interestingly, a prospective study by Dunn et al. based on a cohort of 139 individuals found that preoperative pain catastrophizing, anxiety and depression positively correlated with one another in the modulation of acute postoperative pain, until the patient’s discharge. However, their research did not evaluate the predictive value of the assessed variables but rather correlates of acute postoperative pain. Furthermore, due to the different follow-up period and study design, it is not possible to compare the obtained results with our present findings [34]. Furthermore, several retrospective database studies with multivariate analyses have found that depression and anxiety might be predictors of chronic postoperative pain following cervical [35] or lumbar [36] surgeries or leg pain recurrence in patients after lumbar discectomy [37]. Nevertheless, these studies differ from ours with regard to the patient enrollment procedure and study design (large database analyses versus our prospective cohort study) as well as follow-up period.

In analyzing the potential causes of chronic postoperative pain, it is essential to consider various intraoperative, perioperative, and postoperative factors that influence the clinical outcome in patients with DDD. These include, among others, residual intervertebral disc herniation, insufficient neural structures decompression, nerve damage or the presence of adjacent segment disease, implant migration, screw displacement, and inflammation around the placed instruments [38,39,40,41,42]. Furthermore, several postoperative alterations, such as epidural fibrosis, surgical site infection, postsurgical lumbar facet joint syndrome, and paraspinal muscle, have a proven effect on the persistence of pain following spinal surgery [43,44,45,46,47]. However, as our present study did not include an extended clinical assessment combined with radiological evaluation 12 weeks after the procedure, it is not possible to speculate on the contribution of the above-mentioned factors to the overall assessment of postoperative pain.

Alternatively, elevated postoperative pain levels may be linked to the psychological function of pain. Studies have shown that pain can be a somatic response to experienced stress and may be associated with excessive activation of the immune system [48,49]. In this study, patients with better relationships and support reported lower levels of chronic postoperative pain, suggesting that supportive relationships may alleviate stress levels and, consequently, the intensity of experienced pain. It could be hypothesized that reported pain may encourage the patient to seek closeness and attention in relationships. Nevertheless, this hypothesis requires evaluation in future research. Furthermore, reported complaints may also stem from re-experiencing pain related to trauma. In the absence of access to traumatic memories at the explicit memory level, somatic responses, such as sensory distortions or pain, may be heightened [50]. However, these possibilities should be explored in future studies assessing traumas, dissociations, and somatic responses to stress preoperatively, correlating them with pain levels before and after surgery.

It is important to highlight that in the case of surgical treatment in DDD without neurological deficits, our findings indicate that the most significant benefit may be seen in individuals reporting a preoperative subjective pain level of 6 or above on the VAS before surgery. Patients experiencing lower preoperative pain intensity should consider alternative methods such as physiotherapy [51] or psychotherapy [52], whose effectiveness has been established through randomized controlled trials. Nevertheless, this matter necessitates further investigation.

Our study has certain limitations. First, a relatively low sample size of the study population (N = 81) for logistic regression analysis can reduce the precision of coefficient estimates and increase the likelihood of random error. A relatively low sample size limits the ability to detect genuine relationships between variables and weakens the overall validity and generalizability of the findings. Considering the pilot status of our study, the results can be treated as preliminary, and a larger study needs to be carried out to verify the present results.

Furthermore, the definition of chronic postoperative pain is not standardized in the literature and may refer to varying time frames, such as 6- or 12-months [53,54] following surgery. Also, the examined cohort exhibits heterogeneity within the group (including patients who underwent both lumbar and cervical spine procedures). What is more, the presented analyses lack a preoperative and perioperative standardization regarding analgesic dosing and opioid consumption assessment, as well as a more detailed radiological assessment to evaluate treatment outcomes. Preoperative assessments did not include consultations with physiotherapists or psychotherapists, histories of previous spinal surgeries, or consideration of social factors such as smoking history.

To generalize the obtained results, additional prospective, randomized controlled studies are essential; these should encompass a larger cohort of patients that accurately represent the population undergoing surgical intervention for DDD without neurological deficits. These studies ought to ensure homogeneity by analyzing patients separately, i.e., those undergoing procedures in the cervical and lumbar spine segments should be segregated from those who have previously undergone physiotherapy and psychotherapy for chronic pain. Furthermore, future research could standardize analgesic dosing and evaluate opioid consumption and functional outcome measures such as the Oswestry Disability Index and include a thorough postoperative radiological assessment of treatment outcomes.

## 5. Conclusions

This prospective study with stepwise regression analysis demonstrated that in patients without preoperative neurological deficits, a preoperative pain intensity of 6 or higher on the Visual Analogue Scale (VAS) is an independent predictor of the absence of chronic pain 12 weeks after surgical treatment for degenerative disc disease (DDD). According to the current literature, this is one of only two studies that establish a cut-off point for preoperative pain as a predictor of surgical efficacy and is among the few prospective studies evaluating the predictive value of preoperative pain. Patients who qualified for surgery may also benefit from preoperative consultations with a physiotherapist and a psychotherapist. However, given the complexity of this issue, further prospective, randomized controlled studies are essential.

## Figures and Tables

**Table 1:** Characteristics of the study population: qualitative variables.

Variable	Level	N	%
Sex	Female	48	59.3
	Male	33	40.7
Marital status	Married	53	65.4
	Partnership	12	14.8
	Widow	5	6.2
	Single	11	13.6
Education	Primary	3	3.7
	Bachelor’s/Master’s level	35	43.2
	Vocational	19	23.5
	Secondary	24	29.6
Residence	Other	3	3.7
	Family	71	87.7
	Alone	7	8.6
Place of residence	City of 100–500 thousand	3	3.7
	City of 50–100 thousand	7	8.7
	Town up to 50 thousand	24	30.0
	City over 500 thousand	29	36.3
	Village	17	21.2
Parent	No	16	19.8
	Yes	65	80.2
Student	No	74	91.4
	Yes	7	8.6
Employee	No	21	25.9
	Yes	60	74.1
Previous psychiatric treatment	No	66	81.5
	Yes	15	18.5
Positive family history of mental disorders	No	72	88.9
	Yes	9	11.1
Somatic diseases	No	37	45.7
	Yes	44	54.3
Head injuries	No	69	87.3
	Yes	10	12.7
Regular Use Medicines	No	35	43.2
	Yes	46	56.8
Pain	No	3	3.8
	Yes	77	96.2
Numbness	No	27	34.2
	Yes	52	65.8
Dizziness	No	57	72.2
	Yes	17	21.5
	Yes/No	5	6.3
Weakening of muscle strength	No	73	92.4
	Yes	5	6.3
	Yes/No	1	1.3
Urinary/fecal incontinence	No	74	93.7
	Yes	3	3.8
	Not sure	2	2.5
Number of levels of operation	1	58	75.3
	2	12	15.6
	3	6	7.8
	4	1	1.3

**Table 2:** Relationship of qualitative variables characterizing the study population to the effect of surgery regarding no postoperative improvement in pain after 12 weeks.

Variable	Level	No Pain Improvement After 12 Weeks Since Surgery		Chi^2^ (Yates)	df	*p*
		No N(%)	Yes N(%)			
Sex	Female	30 (56)	14 (70)	0.735	1	0.391
	Male	24 (44)	6 (30)			
Marital status	Married	41 (75.9)	10 (50)	7.126 *	1	0.068
	Relationship	6 (11.1)	4 (20)			
	Widow	1 (1.9)	3 (15)			
	Single	6 (11.1)	3 (15)			
Education	Primary	0 (0)	2 (10)	7.928 *	3	0.048
	Bachelor’s/Master’s level	26 (48.1)	7 (35)			
	Vocational	11 (20.4)	7 (35)			
	Secondary	17 (31.5)	4 (20)			
Residence	Other	1 (1.9)	2 (10)	2.569	2	0.277
	Family	49 (90.7)	17 (85)			
	Alone	4 (7.4)	1 (5)			
Place of residence	City of 100–500 thousand	1 (1.9)	1 (5.3)	2.193 *	4	0.7
	City of 50–100 thousand	6 (11.1)	1 (5.3)			
	Town up to 50 thousand	13 (24.1)	7 (36.8)			
	City over 500 thousand	21 (38.9)	6 (31.6)			
	Village	13 (24.1)	4 (21.1)			
Parent	No	9 (16.7)	5 (25)	0.229	1	0.632
	Yes	45 (83.3)	15 (75)			
Student	No	48 (88.9)	19 (95)	0.123	1	0.726
	Yes	6 (11.1)	1 (5)			
Employee	No	12 (22.2)	7 (35)	0.669	1	0.413
	Yes	42 (77.8)	13 (65)			
Previous psychiatric treatment	No	46 (85.2)	14 (70)	1.316	1	0.251
	Yes	8 (14.8)	6 (30)			
Positive family history of mental disorders	No	48 (88.9)	19 (95)	0.123	1	0.726
	Yes	6 (11.1)	1 (5)			
Somatic diseases	No	26 (48.1)	8 (40)	0.131	1	0.717
	Yes	28 (51.9)	12 (60)			
Head injuries	No	45 (86.5)	17 (85)	<0.001	1	1.0
	Yes	7 (13.5)	3 (15)			
Regular Use Medicines	No	27 (50)	5 (25)	2.768	1	0.096
	Yes	27 (50)	15 (75)			
Pain	No	3 (5.7)	0 (0)	0.181	1	0.67
	Yes	50 (94.3)	20 (100)			
Numbness	No	19 (36.5)	7 (35)	<0.001	1	1.0
	Yes	33 (63.5)	13 (65)			
Number of operation levels >1	No	13 (26)	6 (30)	0.002	1	0.966
	Yes	37 (74)	14 (70)			

Abbreviations: N—number of observations, Chi^2^ (Yates)—Chi^2^ test statistics with the Yates correction for continuity, *—Chi^2^ test statistics, df—degrees of freedom, *p*—test probability (asymptotic, two-tailed).

**Table 4:** Coefficients describing the measures of fit of different logistic regression models.

Model	Deflection	AIC	BIC	df	ΔChi²	*p*	Nagelkerke’s R^2^	R^2^ of Cox and Snell
0	82.48	84.483	86.745	70				
1	64.68	68.684	73.209	69	17.799	<0.001	0.323	0.222
2	60.98	66.981	73.769	68	3.703	0.054	0.38	0.261

Abbreviations: AIC—Akaike Information Criterion, BIC—Bayesian Information Criterion, ΔChi^2^—Chi^2^ statistics change in comparison with the previous model, df—degrees of freedom, *p*—test statistics (two-tailed, asymptotic), R^2^—coefficient of regression.

**Table 5:** Variables included in different models of the forward stepwise logistic regression.

Model	Parameter	Estimate	SE	OR	z	Wald Test			95% CI (OR Scale)	
						Wald Statistics	df	*p*	Lower Limit	Upper Limit
0	(constant)	−1.007	0.268	0.365	−3.756	14.106	1	<0.001	0.216	0.618
1	(constant)	1.623	0.737	5.066	2.202	4.849	1	0.028	1.195	21.47
	VAS_0	−0.508	0.14	0.602	−3.627	13.156	1	<0.001	0.457	0.792
2	(constant)	0.638	0.904	1.892	0.705	0.498	1	0.481	0.322	11.137
	VAS_0	−0.737	0.217	0.478	−3.395	11.525	1	<0.001	0.313	0.732
	WHYMPI—Pain Severity	0.603	0.345	1.828	1.747	3.051	1	0.081	0.929	3.598

Abbreviations: CI—confidence interval, OR—odds ratio, SE—standard error, Z—z statistic—the ratio of the estimated coefficient (regression parameter) to its standard error.

**Table 6:** Preoperative VAS cut-off scores for ROC curve analysis of the discrimination between patients who obtained improvement in pain 12 weeks after the surgical procedure.

Cutpoint for a preoperativeVAS	Sensitivity (%)	Specificity (%)	PPV (%)	NPV (%)	Youden’s J index
0	100%	0%	72.97%	NaN%	0
1	100%	15%	76.06%	100%	0.15
2	98.15%	25%	77.94%	83.33%	0.2315
3	96.30%	40%	81.25%	80%	0.363
4	90.74%	50%	83.05%	66.67%	0.4074
5	81.48%	60%	84.62%	54.55%	0.4148
6	72.22%	75%	88.64%	50%	0.4722
7	53.70%	85%	90.62%	40.48%	0.387
8	42.59%	95%	95.83%	38%	0.3759
9	12.96%	100%	100%	29.85%	0.1296
10	5.56%	100%	100%	28.17%	0.0556

Abbreviations: NaN%—not a number, NPV—negative predictive value, PPV—positive predictive value.

**Table 3:** Differences in quantitative variables between groups, defined by the lack of pain improvement achieved postoperatively, after 12 weeks of observation.

Variable	No Improvement in Pain After 12 Weeks	N	Average	SD	SE	CV	Statistics (df)	*p*	ES
Age	No	47	48.32	12.14	1.771	0.251	0.712 ^a^ (76)	0.478	-
	Yes	31	46.29	12.57	2.258	0.272			
Years of education	No	47	13.49	2.77	0.403	0.205	606.5 ^b^	0.198	-
	Yes	31	14.42	3.29	0.592	0.228			
SBP	No	47	128.2	43.33	6.32	0.338	946 ^b^	0.027	0.299
	Yes	31	122.2	35.36	6.35	0.289			
DBP	No	47	81.4	27.7	4.04	0.34	874 ^b^	0.138	-
	Yes	31	78.23	22.39	4.022	0.286			
Preoperative VAS	No	47	6.915	2.02	0.295	0.292	1203 ^b^	<0.001	0.651
	Yes	31	4.032	2.331	0.419	0.578			
Duration of pain before surgery [days]	No	40	182.2	288.7	45.47	1.585	536.5 ^b^	0.833	-
	Yes	26	202.04	295.4	57.93	1.462			
Duration of the surgical procedure [min]	No	32	154.5	72.55	12.83	0.469	406 ^b^	0.929	-
	Yes	25	152.4	65.94	13.19	0.433			
HADS-A	No	47	7.915	3.889	0.567	0.491	0.127 ^a^ (76)	0.899	-
	Yes	31	8.032	4.135	0.743	0.515			
HADS-D	No	47	5.468	3.629	0.529	0.664	1.569 ^a^ (76)	0.121	-
	Yes	31	6.935	4.604	0.827	0.515			
PSS-10	No	45	21.89	6.001	0.895	0.274	0.915 ^a^ (73)	0.363	-
	Yes	30	20.47	7.399	1.351	0.361			
FCB-TI—Perseverance	No	45	11.778	4.122	0.615	0.350	1.446 ^a^ (73)	0.152	-
	Yes	30	13.200	4.246	0.775	0.322			
FCB-TI—Sensory Sensitivity	No	44	14.614	3.021	0.455	0.207	1.441^a^ (72)	0.154	-
	Yes	30	13.600	2.896	0.529	0.213			
FCB-TI—Reactivity	No	44	9.636	4.808	0.725	0.499	1.445 ^a^ (72)	0.153	-
	Yes	30	11.333	5.175	0.945	0.457			
FCB-TI—Endurance	No	44	9.068	4.702	0.709	0.519	0.961 ^a^ (72)	0.34	-
	Yes	30	8.000	4.683	0.855	0.585			
FCB-TI—Activity	No	44	8.250	4.064	0.613	0.493	0.202 ^a^ (72)	0.84	-
	Yes	30	8.467	5.144	0.939	0.608			
FCB-TI—Briskness	No	44	14.886	3.558	0.536	0.239	692.5 ^b^	0.723	-
	Yes	30	14.400	3.997	0.730	0.278			
NEO-FFI—Neuroticism	No	45	19.36	6.800	1.014	0.351	1.887 ^a^ (73)	0.063	-
	Yes	30	22.67	8.327	1.520	0.367			
NEO-FFI—Extraversion	No	45	27.67	4.763	0.710	0.172	805 ^b^	0.16	-
	Yes	30	26.67	6.440	1.176	0.241			
NEO-FFI—Openness to experience	No	45	24.62	4.988	0.744	0.203	613 ^b^	0.716	-
	Yes	30	25.50	5.211	0.951	0.204			
NEO-FFI—Agreeableness	No	45	32.44	5.030	0.750	0.155	0.394 ^a^ (73)	0.695	-
	Yes	30	31.97	5.308	0.969	0.166			
NEO-FFI—Conscientiousness	No	45	33.87	7.219	1.076	0.213	822 ^b^	0.113	-
	Yes	30	29.90	9.342	1.706	0.312			
WHYMPI—Perceived Interference	No	45	3.553	1.308	0.195	0.368	0.959 ^a^ (73)	0.341	-
	Yes	30	3.258	1.295	0.236	0.397			
WHYMPI—Support	No	45	5.227	0.889	0.133	0.170	861 ^b^	0.041	0.276
	Yes	30	4.761	1.062	0.194	0.223			
WHYMPI—Pain Severity	No	45	3.965	1.297	0.193	0.327	887 ^b^	0.022	0.314
	Yes	30	3.288	1.320	0.241	0.402			
WHYMPI—Perceived Life Control	No	45	4.222	1.475	0.220	0.349	700.5 ^b^	0.785	-
	Yes	30	4.259	1.099	0.201	0.258			
WHYMPI—Affective Distress	No	45	3.298	1.125	0.168	0.341	1.799 ^a^ (73)	0.076	-
	Yes	30	2.815	1.159	0.212	0.412			
WHYMPI—Negative Responses	No	45	0.956	1.094	0.163	1.145	686 ^b^	0.907	-
	Yes	30	1.120	1.527	0.279	1.363			
WHYMPI—Solicittous Responses	No	45	4.569	1.114	0.166	0.244	927.5 ^b^	0.006	0.374
	Yes	30	3.574	1.501	0.274	0.420			
WHYMPI—Distracting Responses	No	45	3.090	1.160	0.173	0.375	2.441^a^ (73)	0.017	0.575
	Yes	30	2.357	1.431	0.261	0.607			
WHYMPI—Household Chores	No	45	3.527	1.640	0.244	0.465	659 ^b^	0.867	-
	Yes	30	3.580	1.732	0.316	0.484			
WHYMPI—Outdoor Work	No	45	1.698	1.811	0.270	1.067	585.5 ^b^	0.334	-
	Yes	30	1.870	1.526	0.279	0.816			
WHYMPI—Activities Away from Home	No	45	2.572	1.364	0.203	0.530	0.132 ^a^ (73)	0.895	-
	Yes	30	2.617	1.511	0.276	0.577			
WHYMPI—Social Activity	No	45	2.694	1.198	0.179	0.444	0.143 ^a^ (73)	0.887	-
	Yes	30	2.734	1.138	0.208	0.416			
WHYMPI—General Activity	No	45	2.619	1.090	0.163	0.416	615 ^b^	0.52	-
	Yes	30	14.624	45.236	8.259	3.093			
SSS-8	No	47	12.02	5.765	0.841	0.48	0.695 ^a^ (76)	0.489	-
	Yes	31	11.13	5.188	0.932	0.466			
TMT-A time	No	47	37.787	14.929	2.178	0.395	874 ^b^	0.138	-
	Yes	31	32.452	15.281	2.745	0.471			
TMT-A count error	No	47	0.277	0.971	0.142	3.512	719 ^b^	0.881	-
	Yes	31	5.903	23.758	4.267	4.025			
TMT-B time	No	47	82.383	29.464	4.298	0.358	856 ^b^	0.193	-
	Yes	31	72.419	34.318	6.164	0.474			
TMT-B count error	No	47	1.596	4.052	0.591	2.539	732 ^b^	0.969	-
	Yes	31	0.968	2.089	0.375	2.159			
DSF	No	47	5.340	0.984	0.144	0.184	815 ^b^	0.360	-
	Yes	31	5.065	1.093	0.196	0.216			
DSB	No	47	3.894	0.961	0.140	0.247	763 ^b^	0.712	-
	Yes	31	3.839	0.969	0.174	0.253			

Abbreviations: a—Student’s *t*-test, b—Mann–Whitney U-test, CV—coefficient of variation, DBP—diastolic blood pressure, df—number of degrees of freedom, DSB—Digit Span Backwards, DSF—Digit Span Forwards, ES—effect size, FCB-TI—The Formal Characteristics of Behavior–Temperament Inventory, HADS-A—Hospital Anxiety and Depression Scale—Anxiety, HADS-D—Hospital Anxiety and Depression Scale—Depression, N—population size, *p*—test probability (asymptotic, two-tailed), NEO-FFI—NEO Five-Factor Inventory, PSS-10—Perceived Stress Scale 10, SBP—systolic blood pressure, SD—standard deviation, SE—standard error, SSS-8—Somatic Symptoms Scale 8, TMT—Trail Making Test A, TMT-B—Trail Making Test B, VAS—Visual Analogue Scale, WHYMPI—West Haven-Yale Multidimensional Pain Inventory.

## Data Availability

The datasets generated and analyzed in the current study are available from the corresponding author upon reasonable request.

## Supplementary Materials

The following supporting information can be downloaded at: https://www.mdpi.com/article/10.3390/jcm14103467/s1, Table S1: Characteristics of the study population: quantitative variables; Table S2: Characteristics of the study population: quantitative variables, cont.
