# Supplementary material for: The Preoperative Level of Pain Predicts Chronic Pain in Patients Operated on for Degenerative Disc Disease—A Prospective Study
Source: J Clin Med. 2025 May 15;14(10):3467. doi: 10.3390/jcm14103467 (PMC12112273; doi:10.3390/jcm14103467)
Supplement: Supplementary file 1 [file jcm-14-03467-s001.zip › jcm-3638512-supplementary.pdf]

Supplementary Data

Table S1. Characteristics of the study population: quantitative variables.

Variable	Median	Mean	SD	IQR	Shapiro-Wilk	Shapiro-Wilk p-value	Min	Max
Age [years]	47	47.407	12.054	18	0.973	0.088	20	69
Education [years]	13	13.914	2.976	6	0.867	< .001	8	20
SBP [mmHg]	133	125.543	39.475	20	0.684	< .001	0	220
DBP [mmHg]	85	80.086	25.132	13	0.691	< .001	0	130
Duration of the surgical procedure [min]	137.5	153.167	69.422	81.25	0.936	0.004	45	330
Duration of pain before the procedure [days]	60	182.522	285.038	172	0.65	< .001	0	1095
Preoperative VAS	6	5.728	2.574	4	0.948	0.002	0	10
SSS-8	11	11.728	5.436	7	0.97	0.051	0	25
TMT - A time [s]	35	35.444	15.189	17	0.921	< .001	0	97
TMT - A count error	0	2.42	14.826	0	0.155	< .001	0	121
TMT - B time [s]	71	78.691	31.751	37	0.962	0.016	0	161
TMT - B count error	0	1.42	3.485	1	0.478	< .001	0	20
DSF	5	5.222	1.025	2	0.91	< .001	3	8
DSB	4	3.889	0.962	1	0.9	< .001	2	7
HADS-A	8	7.938	3.992	6	0.982	0.328	0	17
HADS-D	6	6.296	4.619	6	0.915	< .001	0	26
PSS-10	23	21.487	6.57	6	0.892	< .001	0	36

Abbreviations: DBP - diastolic blood pressure, DSB - Digit Span Backwards, DSF - Digit Span Forwards, HADS-A - Hospital Anxiety and Depression Scale - Anxiety, HADS-D - Hospital Anxiety and Depression Scale - Depression, IQR – interquartile range, Max – maximum score, Min – minimum score, PSS-10 - Perceived Stress Scale - 10, SBP - systolic blood pressure SD – standard deviation of the mean, SSS-8 - Somatic Symptoms Scale - 8, TMT - Trail Making Test.

Table S2. Characteristics of the study population: quantitative variables, cont.

Variable	Median	Mean	SD	IQR	Shapiro-Wilk	Shapiro-Wilk p-value	Min	Max
FCB-TI - Perseverance	12	12.346	4.288	6.75	0.976	0.15	0	21
FCB-TI - Sensory Sensitivity	14	14.377	3.07	4	0.956	0.009	5	20
FCB-TI - Reactivity	10	10.286	4.965	7	0.979	0.231	0	20
FCB-TI - Endurance	8	8.701	4.727	8	0.967	0.042	0	18
FCB-TI - Activity	8	8.377	4.422	6	0.963	0.024	1	18
FCB-TI - Briskness	15	14.779	3.709	4	0.942	0.002	5	20
NEO-FFI - Neuroticism	20	20.5	7.49	10.75	0.988	0.694	0	40
NEO-FFI - Extraversion	26	27.282	5.458	8	0.983	0.379	15	42
NEO-FFI - Openness to experience	25	25.231	5.24	5.75	0.973	0.096	9	36
NEO-FFI - Agreeableness	33	32.308	5.145	6.75	0.975	0.131	22	43
NEO-FFI - Conscientiousness	33.5	32.513	8.247	9.25	0.948	0.003	3	47
WHYMPI - Perceived Interference	3.497	3.447	1.284	1.528	0.978	0.185	0	5.667
WHYMPI - Support	5.333	5.068	0.974	1.667	0.855	< .001	2.6	6
WHYMPI - Pain Severity	3.835	3.692	1.335	1.917	0.945	0.002	0	6
WHYMPI - Perceived Life Control	4	4.234	1.319	1.875	0.925	< .001	0	6
WHYMPI - Affective Distress	3.333	3.104	1.146	1.333	0.985	0.472	0	6
WHYMPI - Negative Responses	0.5	0.985	1.266	1.5	0.774	< .001	0	5.6
WHYMPI - Solicittous Responses	4.333	4.204	1.351	1.667	0.94	0.001	0	6
WHYMPI - Distracting Responses	3	2.804	1.343	2	0.972	0.089	0	5.2

WHYMPI - Household Chores	3.6	3.563	1.64	2.55	0.957	0.011	0	6
WHYMPI - Outdoor Work	1.4	1.771	1.681	2.5	0.881	< .001	0	6
WHYMPI - Activities Away from Home	2.5	2.599	1.425	2.125	0.965	0.03	0	5
WHYMPI - Social Activity	2.75	2.699	1.152	1	0.967	0.04	0	5.5
WHYMPI - General Activity	2.861	7.243	28.388	1.3	0.172	< .001	0	182

Abbreviations: FCB-TI - The Formal Characteristics of Behavior-Temperament Inventory, IQR – interquartile range, Max – maximum score, Min – minimum score, NEO-FFI - NEO Five-Factor Inventory, SD – standard deviation of the mean, WHYMPI - West Haven-Yale Multidimensional Pain Inventory
